# Research status and trends of enterprise safety culture: a knowledge graph analysis based on CiteSpace

**DOI:** 10.3389/fpubh.2024.1362830

**Published:** 2024-03-07

**Authors:** Ming Wen, Zhaohui Gou, Chaogang Xiong, Yao Wang, Dan Cheng

**Affiliations:** ^1^Safety, Environment and Technology Supervision Research Institute of PetroChina Southwest Oil and Gas Field Company, Chengdu, China; ^2^PetroChina Southwest Oil and Gas Field Company, Chengdu, China

**Keywords:** safety culture, knowledge graph analysis, enterprise safety culture, citation burst, CiteSpace

## Abstract

The cultivation of safety culture within enterprises has evolved into a pivotal facet of overall safety progress, drawing increased attention from a myriad of businesses. With a comprehensive examination of relevant literature, 635 documents from both domestic and international sources were selected as the subjects of analysis. The developmental trends, both domestically and internationally, follow a generally consistent pattern, resembling an inverted “V” shape. The initial phase witnessed gradual development, followed by a substantial and rapid growth phase in the mid-term. In the later phase, a decline is observed. This study utilizes the CiteSpace software for keyword clustering analysis, employing the Log Likelihood Ratio (LLR) algorithm with default parameters to delve into the themes within the specific research field of enterprise safety culture. It was observed that domestic research predominantly centers on the practical perspective of mitigating accidents through the establishment of enterprise safety culture, while international research places greater emphasis on theoretical considerations, specifically focusing on the impact of safety culture atmospheres within enterprises on employees.

## 1 Introduction

Following the Chernobyl nuclear accident in 1986, the introduction of the “safety culture” triggered extensive discussions within the global safety science community. Aligned with the contemporary safety management philosophy, tenets such as “safety first,” “people-oriented,” and “care for life, focus on safety” have been seamlessly integrated into safety culture construction, emerging as indispensable guiding principles for enterprise safety management. The cultivation of safety culture within enterprises has evolved into a pivotal facet of overall safety progress, drawing increased attention from a myriad of businesses. Yet, in the course of safety culture construction, enterprises have grappled with a lack of comprehension and recognition regarding the interplay between safety culture and safety management. Challenges have arisen, exemplified by perspectives like “culture is useless,” treating safety culture construction as mere rhetoric or the posting of slogans. Furthermore, some have disconnected safety culture construction from practical safety management, impeding enterprises from achieving safety management enhancement through safety culture initiatives.

In recent years, both domestic and international scholars have conducted extensive research on the connotations, characteristics, dimensions, measurement methods, and influencing factors of enterprise safety culture (1–4). Through a comprehensive review and analysis of abundant literature, it is evident that domestic research on enterprise safety culture predominantly centers on conceptual definition, dimension delineation, measurement methods, and influencing factors (5, 6). In contrast, international research places a greater emphasis on theoretical exploration (7, 8). Due to disparities in cultural backgrounds, economic levels, and developmental stages between domestic and international countries, domestic scholars focus on comparative studies among different nations (9). However, the existing theoretical research on enterprise safety culture in domestic remains in an exploratory stage, necessitating further enrichment and refinement. This review and analysis of relevant literature, both domestic and international, aim to furnish a reference for the theoretical and practical research on enterprise safety culture in domestic.

## 2 Literature retrieval of enterprise safety culture

### 2.1 Definition of enterprise safety culture

The notion of enterprise safety culture was initially introduced by safety management personnel. Despite sustained research interest in recent years, a unified consensus on its conceptual definition remains elusive. Presently, three primary perspectives contribute to the understanding of the content of enterprise safety culture. Firstly, safety culture is perceived as a people-oriented management philosophy, accentuating the pivotal role of individuals in the production process, thereby establishing an innovative management model. This culture-oriented perspective is chiefly evident in employees’ attitudes and behaviors toward safety production (10). Secondly, safety culture encompasses the values, behavioral norms, regulatory systems, and management methods shaped by enterprises in the course of production and operation. Its core content embodies a management philosophy and ideology developed by enterprises concerning people, objects, environment, and management throughout the production and operation process (11). Thirdly, safety culture is regarded as a novel type resulting from the integration of safety management into enterprise culture construction, guided by safety management theory. This emerging culture emphasizes commencing with people, conducting comprehensive management and control of individuals throughout the entire process, encompassing the cultivation of safety awareness, and guiding behavior norms (12).

Consequently, in this study, during the literature selection process, a comprehensive consideration of the three aforementioned aspects is undertaken to define the concept of enterprise safety culture. Further distinctions are made in researching specific themes during the literature screening process.

### 2.2 Data sources

(1) Retrieval sources: The Web of Science and CNKI databases served as the chosen retrieval sources. Employing a standardized sampling method, the literature’s validity was affirmed through three rules: First, screening for literature relevance based on research topics and abstracts; Second, deduplication of all retrieval results; Third, reading the content of the remaining literature, leading to the exclusion of irrelevant materials. This meticulous process yielded a total of 635 valid retrieval results (refer to Table 1 for details).
(2) Keywords retrieval: The selection of keywords represents a critical and challenging aspect of literature research. The significance lies in the fact that keyword selection shapes the research content and direction, while the challenge arises from the varying perspectives of different academic factions. Initially searching using “enterprise safety culture” and “*企业安全文化*(enterprise safety culture)” as subject terms in selected domestic and international journals, the preliminary search results underwent careful review to eliminate articles with lower relevance to the degree of enterprise safety culture, such as those related to safety education, food safety, medical safety, etc. The research sample for this study was then meticulously determined.
(3) Timespan: To secure a comprehensive research sample, the time range for literature retrieval was extended to the years 1900–2023. The final retrieval results indicated that formal literature on foreign enterprise safety culture research can be traced back to 2007 with the publication of “Risk, safety and culture in Brazil and Argentina: the case of TransInc Corporation” by Perez-Floriano and Gonzalez. The earliest domestic article on enterprise safety culture research was Professor Cao Qi’s discussion on the category of safety culture published in 1995 in “Labor Protection.”

**Table 1:** Literature retrieval.

Database	Keyword	Document type	Conditions	Timespan	Records	Qualified
CNKI	企业&安全文化(enterprise & safety culture)	主题(topic)	1. “类别”选择“学术期刊”(Document type—academic journal)	1900–2023	697	567
			2. “来源类别”选择“核心期刊”(citing source—core collection)			
Web of Science	Enterprise safety culture	topic	1. “Database”—“Web of Science Core Collection”	1900–2023	179	68
			2. “Document type”—“article、review article、published online”			
Article included finally						635

To avoid overlooking relevant literature, this study retained literature that clearly included the aforementioned keywords in the title or abstract fields. Additionally, through literature tracing, the indexing and citation status of the selected literature were investigated. With this retrieval strategy, this study ensured the inclusion of important literature, ultimately selecting 635 research samples, including 68 foreign-language articles and 567 domestic articles, forming the initial sample for this paper.

### 2.3 Research trends

**Figure 1:**
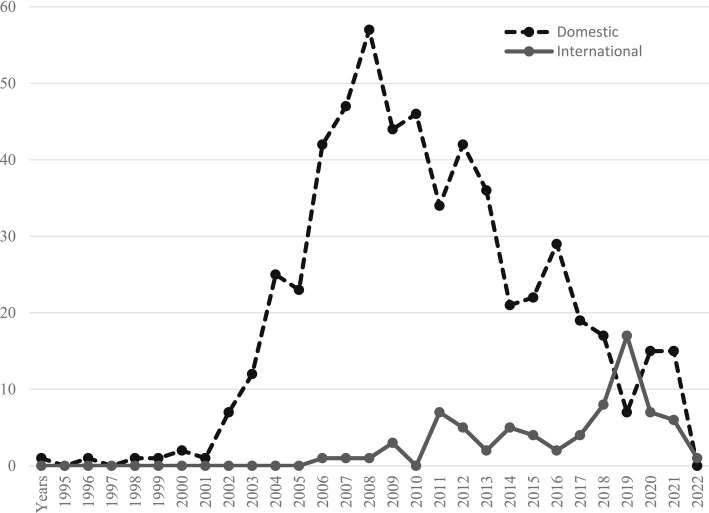
Trends in domestic and international publications on enterprise safety culture.

Through literature retrieval and screening, this study conducted a comprehensive analysis of the literature sources. The developmental trends, both domestically and internationally, follow a generally consistent pattern, resembling an inverted “V” shape. The initial phase witnessed gradual development, followed by a substantial and rapid growth phase in the mid-term. In the later phase, a decline is observed, but there is a notable difference in the overall research quantity (Figure 1).

International research commenced relatively late, with the first article appearing in 2007. It can be broadly divided into three stages. The first stage, spanning from 2007 to 2017, represents the initial development phase characterized by slow growth, with only 1–5 articles per year. During this period, there was limited relevant research, and the outcomes were not yet mature. The second stage, from 2018 to 2020, witnessed accelerated growth, with the number of articles surging from 8 in 2019 to 17 in 2020, indicating an intensified growth rate. The third stage, from 2021 to 2023, reveals a growth decline, returning to an annual publication quantity of around 5 articles, with a more measured growth rate.

Contrastingly, domestic research initiated earlier and compared to international research, demonstrates an overall trend of “rapid growth and stable development.” It can be roughly divided into three stages. The first stage, from 1995 to 2002, represents a period of stable development, with an annual publication quantity of around 1 article, indicating limited relevant research. The second stage, from 2003 to 2009, is characterized by rapid growth, exhibiting the fastest growth rate, increasing consistently each year. The annual number of published articles remained between 30 and 50, peaking at 57 in 2009. The third stage, from 2009 to 2023, signifies a growth decline, with a decreasing growth rate and an annual average publication quantity maintained at 15 to 25 articles.

## 3 Methods

This paper chooses CiteSpace as a knowledge graph analysis tool to examine research related to corporate safety culture, and its rationality lies in the following three aspects.

Firstly, corporate safety culture is a complex and multidimensional research field that involves multiple aspects such as safety values, safety thinking and awareness, safety style and attitude, safety management mechanisms and norms. These aspects are interrelated and mutually influential, forming a complex knowledge network. CiteSpace, as a professional knowledge graph analysis tool, can deeply explore and analyze these complex knowledge networks, helping researchers better understand and grasp the connotation and extension of corporate safety culture.

Secondly, research on corporate safety culture requires a large amount of literature support and empirical analysis. CiteSpace has powerful literature data processing capabilities, which can quickly and accurately extract key information from literature, such as authors, institutions, keywords, citation relationships, etc., thereby helping researchers build a complete and accurate knowledge graph. At the same time, CiteSpace also supports various visual display methods, such as clustering graphs, timeline graphs, network graphs, etc., allowing researchers to have a more intuitive understanding of the research status and development trends of corporate safety culture.

Finally, research on corporate safety culture needs to be constantly updated and improved. With the continuous development of society and the continuous innovation of enterprise safety management, the connotation and extension of enterprise safety culture are also constantly changing. CiteSpace has powerful dynamic updating capabilities, which can track the latest research results and dynamic changes in real time, helping researchers to timely understand and grasp the latest progress and trends in corporate safety culture research.

However, CiteSpace may be affected by factors such as incomplete data sources, language and cultural biases, and differences in user interpretation and understanding during its application, making it difficult for research results to truly reflect the current status and trends in the field of safety culture. Based on the limitations of Citespace itself mentioned above, this study comprehensively considered Chinese and English literature databases to ensure sufficient literature coverage. At the same time, the research team has participated in safety culture related research and practice activities in various countries around the world multiple times, reducing differences in understanding of safety culture in enterprises from different countries. In addition, due to the long-term work of the research team in the field of safety culture, Have a certain level of professional knowledge and the ability to explain research trends. Therefore, it can effectively enhance the adaptability of research methods and improve the scientificity of research conclusions.

The CiteSpace V software utilized in this study is a widely used tool for scientific literature metrics. Developed by Professor Chaomei Chen, a Chinese American scholar at Drexel University in the United States, this analysis tool is based on the Java environment. Introduced to China in 2006, the software is extensively employed in both natural and social sciences, renowned for its notable feature of visualizing literature metrics. Its application extends to the analysis of literature, identification of research development trajectories, exploration of cutting-edge directions, and highlighting of hot topics. The software enables the quantitative and dynamic presentation of evolutionary trends and the current status within a research field.

### 3.1 Keywords clustering analysis

**Figure 2:**
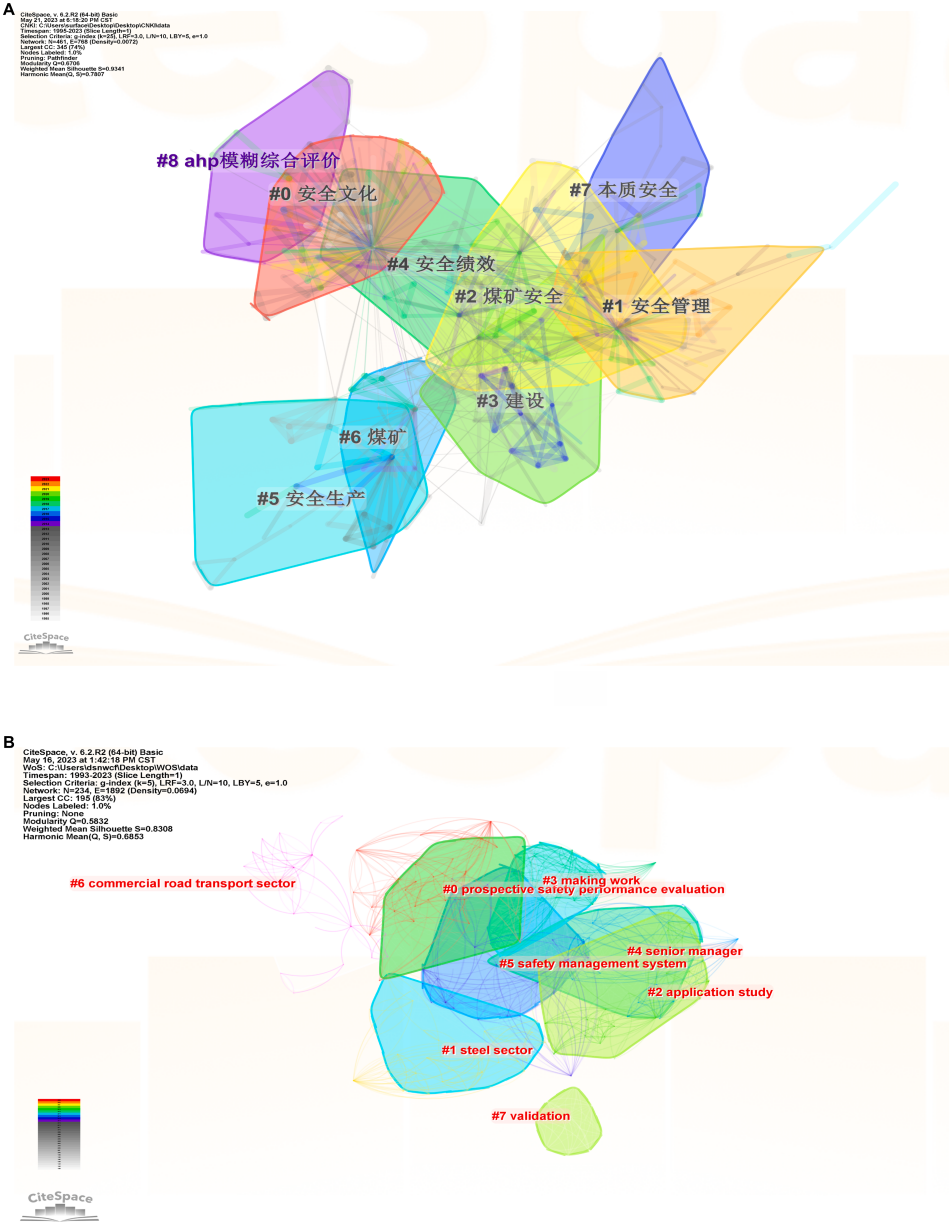
Clustering map of keywords on enterprise safety culture between domestic and international. **(A)** for domestic and **(B)** for international. In the **(A)**, #0 = safety culture, #1 = safety management, #2 = coal mine safety, #3 = construction, #4 = safety performance, #5 = safety production, #6 = coal mine, #7 = intrinsic safety, #8 = AHP fuzzy comprehensive evaluation.

This study utilizes the CiteSpace software for keyword clustering analysis, employing the Log Likelihood Ratio (LLR) algorithm with default parameters to delve into the themes within the specific research field of enterprise safety culture. The Modularity Q values, assessing network structure and clustering clarity, stand at 0.6706 for domestic research and 0.5832 for international research. These values surpass the standard threshold (Q ≥ 0.3), indicating a robust clustering structure and high network stability. Additionally, the Mean Silhouette (S value) scores of 0.9341 and 0.8308 for domestic and international research, respectively, exceed the general standard (S ≥ 0.7), signifying excellent clustering effects with notable homogeneity, effective clustering, and clear thematic delineation (Figure 2).

Examining the clustering results, domestic enterprise safety culture research unfolds into nine distinct themes, with primary focuses on “safety culture” and “safety management.” Concurrently, attention is directed toward safety performance and case studies in coal mine safety. In the international domain, enterprise safety culture research crystallizes into eight themes, spotlighting “prospective safety performance evaluation,” “application study,” and “safety management system.” Noteworthy is the frequent utilization of coal mine safety as a case study in international research, unraveling the intricacies of safety culture through the analysis of incidents and addressing deficiencies in enterprise safety culture.

### 3.2 Keyword frequency and centrality analysis

To gain deeper insights into research hotspots within enterprise safety culture and explore evolving trends, this study employs co-occurrence and timeline analyses of keywords in the literature collection. Research hotspots signify topics commonly addressed by scholars, requiring a quantitative evaluation of centrality to gage keyword significance within the co-word network, considering network science principles.

**Figure 3:**
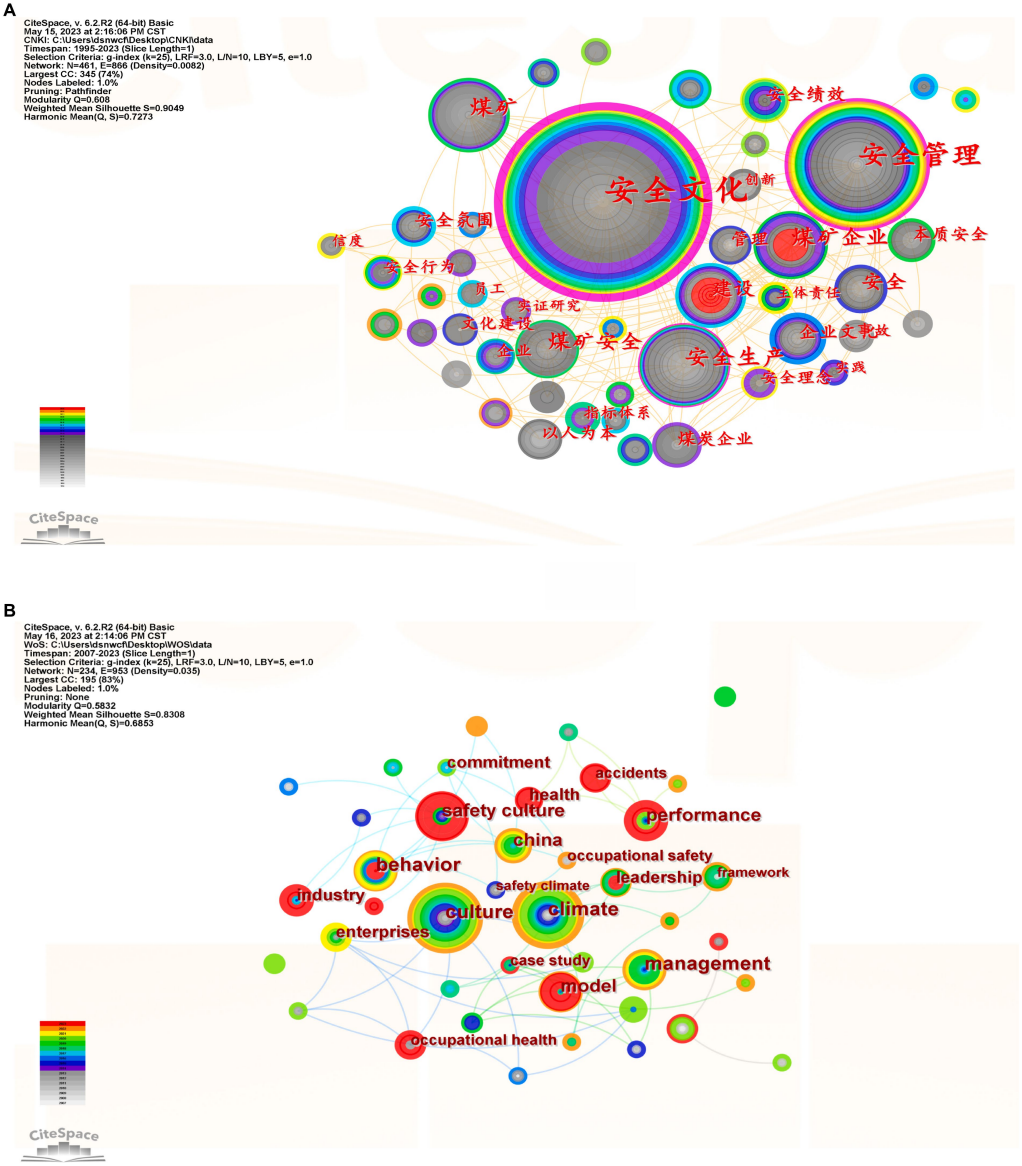
Map of keywords co-occurrence on enterprise safety culture between domestic and international. **(A)** for domestic and **(B)** for international.

**Figure 4:**
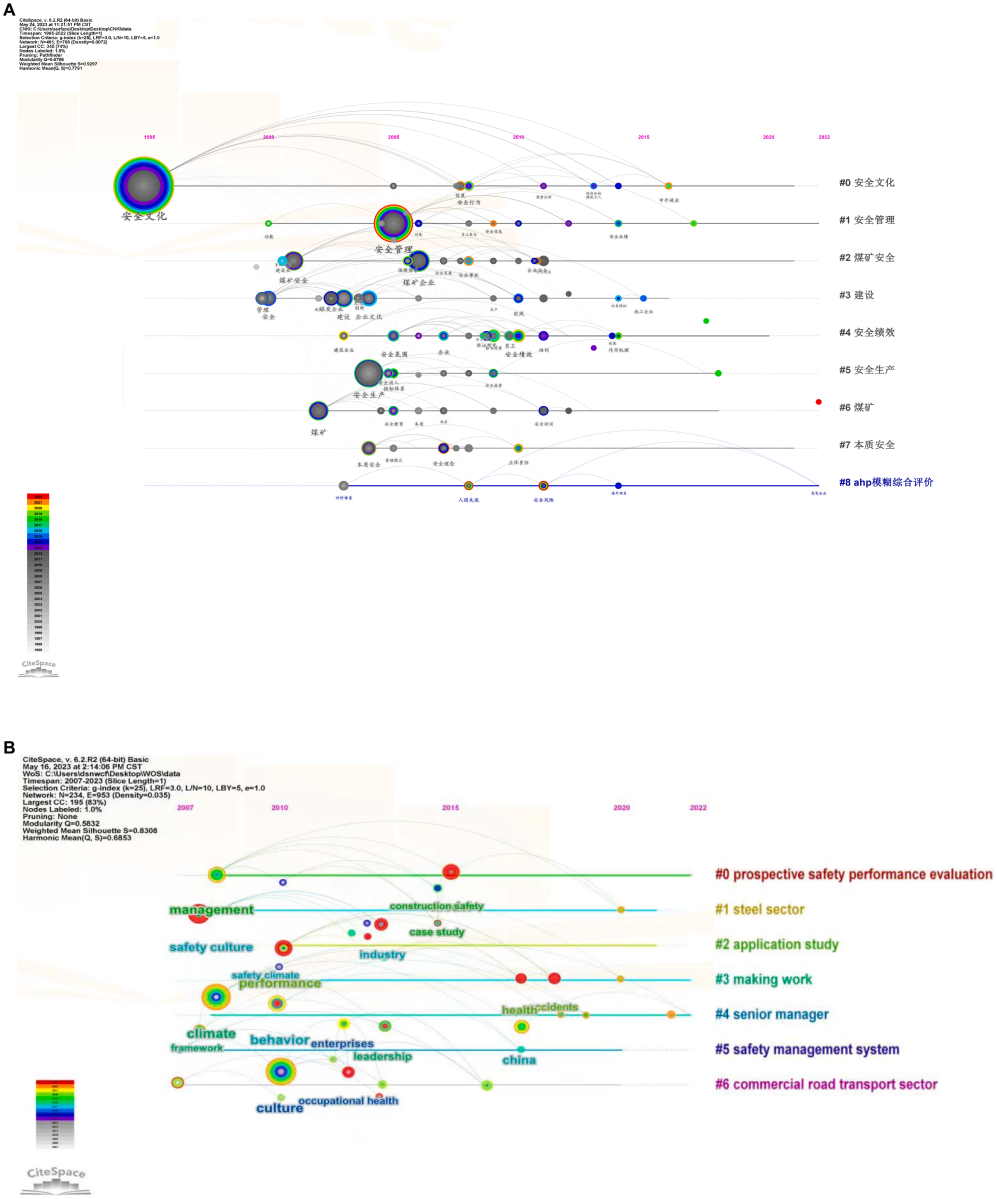
Timeline map of the evolution of keywords on enterprise safety culture. **(A)** for domestic and **(B)** for international. In the **(A)**, #0 = safety culture, #1 = safety management, #2 = coal mine safety, #3 = construction, #4 = safety performance, #5 = safety production, #6 = coal mine, #7 = intrinsic safety, #8 = AHP fuzzy comprehensive evaluation.

The visualization software CiteSpace V constructs a knowledge graph of keyword clusters, analyzing the knowledge structure and research directions in the field of enterprise safety culture. The CiteSpace analysis model spans “1995–2023” and “2007–2023,” extracting keywords yearly. CiteSpace generates a keyword co-occurrence graph (Figure 3), identifying high-frequency keywords with centrality (Tables 2, 3, displaying keywords with a frequency greater than 2 and centrality exceeding 0.01). Figure 4 illustrates that both domestic and international research exhibits a multi-axis radial structure converging toward the center. The co-occurrence graph for domestic research comprises 461 nodes and 866 connections with a density of 0.0082, while the international counterpart has 234 nodes and 953 connections with a density of 0.035. The overall low density in both contexts suggests a relatively divergent landscape in current research on enterprise safety culture.

**Table 2:** The keywords and centrality in the domestic research field of enterprise safety culture from 1995 to 2023.

Rank	Keywords	Frequency	Centralities
1	安全文化(safety culture)	202	0.81
2	安全管理(safety management)	78	0.25
3	煤矿(coal mine)	37	0.03
4	安全生产(safety production)	35	0.11
5	煤矿企业(coal mine enterprise)	26	0.08
6	煤矿安全(coal mine safety)	24	0.04
7	建设(construction)	20	0.01
8	企业文化(enterprise culture)	16	0.02
9	安全(safety)	15	0.03
10	本质安全(intrinsic safety)	13	0.04
11	煤炭企业(coal enterprise)	13	0.01
12	安全绩效(safety performance)	12	0.01
13	以人为本(people-oriented)	11	0.02
14	安全氛围(safety atmosphere)	11	0.03
15	企业(enterprise)	10	0.01
16	安全行为(safety behavior)	10	0.02

**Table 3:** The keywords and centrality in the international research field of enterprise safety culture from 2007 to 2023.

Rank	Keywords	Frequency	Centralities
1	Culture	24	0.23
2	Climate	23	0.17
3	Safety culture	16	0.18
4	Behavior	13	0.17
5	Performance	13	0.21
6	Management	13	0.22
7	Model	11	0.12
8	Industry	8	0.04
9	Leadership	8	0.04
10	Accidents	7	0.04
11	Health	7	0.05
12	Occupational health	7	0.03
13	China	6	0.13
14	Risk management	5	0.09
15	Enterprises	5	0.1
16	Framework	5	0.02

In the co-occurrence graph, the positive correlation between keyword frequency and centrality reflects scholars’ collective attention, unveiling research hotspots. Centrality signifies a node’s significance, with higher frequency and centrality indicating greater importance in the research domain. A comparison of domestic and international research exposes variations in focus areas and keywords, suggesting distinct research intensities and frequencies.

For domestic research (Table 2), keywords occurring 10 times or more total 16, with the top ten including safety culture (202 times, 0.81), safety management (78 times, 0.25), coal mine (37 times, 0.03), safety production (35 times, 0.11), coal mine enterprise (26 times, 0.08), coal mine safety (24 times, 0.04), construction (20 times, 0.01), enterprise culture (16 times, 0.03), safety (15 times, 0.03), intrinsic safety (13 times, 0.04). Frequent mentions of safety highlight its centrality, serving as a crucial “intermediary” term in enterprise safety culture research. “High-frequency but low-centrality” keywords like “coal mine” and “coal mine safety” suggest their importance is relatively low despite frequent references, possibly due to a narrowed focus on specific aspects of enterprise safety culture, such as energy industry concerns.

In foreign research (Table 3), keywords occurring 5 times or more total 16, with the top ten including culture (24 times, 0.03), climate (23 times, 0.17), safety culture (16 times, 0.08), behavior (13 times, 0.17), performance (13 times, 0.21), management (13 times, 0.22), model (11 times, 0.12), industry (8 times, 0.04), leadership (8 times, 0.04), accidents (7 times, 0.04). The frequent mention of “culture” demonstrates high centrality in the knowledge graph, serving as a significant term in enterprise safety culture research. However, “culture” and “safety culture” are “high-frequency but low-centrality” keywords, indicating their relatively lower importance in the field. This may stem from less related knowledge in foreign research, gradually analyzed and researched by scholars since 2007, resulting in fewer theoretical achievements.

### 3.3 Timeline evolution analysis of keywords

Utilizing CiteSpace software to construct a timeline graph of keywords in the field of enterprise safety culture unveils the duration and evolution trends of research hotspots, depicted in Figure 4. The chronological arrangement of the keyword evolution graph, from left to right, showcases square nodes whose sizes are proportional to the frequency of corresponding keywords. Conducting a thematic evolution analysis of research hotspots in both domestic and foreign selected literature using CiteSpace software, with time parameters set as 1995–2023 and 2007–2023, Year per slice = 1, Node type = Keyword, the results of the thematic cluster evolution are illustrated in the graph. The graph generated from domestic literature produces 461 nodes and 768 links, with *Q* = 0.6706, while the graph generated from foreign literature produces 234 nodes and 953 links, with *Q* = 0.5832, indicating a clearly defined structure.

Examining the timeline evolution graph of keywords, the prominence of keywords in the domestic timeline has undergone two significant phases with substantial fluctuations: A large-span stage with considerable fluctuations and a larger-span stage with an increase followed by a decrease. This indicates that the development of research on enterprise safety culture in domestic has experienced three stages: steady development, rapid growth, and relatively stable development. Domestic researchers have encountered certain fluctuations in the process of studying enterprise safety culture.

In the graph, besides the node with the maximum frequency for the term “safety culture,” two other significant nodes, “safety management” and “safety production,” emerged around 2005. This signifies that these terms are research hotspots in this field, indicating the importance of safety management and safety production in the development of enterprise safety culture research. The prominence of the keyword “safety production” takes the forefront of the timeline, suggesting that research in this field began exploring based on this keyword, and subsequently, all related studies on enterprise safety culture are associated with this keyword.

This emergence can be attributed to the surge in safety accidents in domestic in 2005, with an increase of 34.9% YoY in severe accidents in coal mining enterprises. The State Council responded by issuing special regulations on preventing coal mine production safety accidents that year (13). This led to a systematic analysis of the causes of frequent production accidents and targeted improvements in the state of production safety, making safety production and safety management hot topics in the research on enterprise safety culture. On the right side of the graph are keyword cluster labels, evolving into nine clusters: safety culture, safety management, coal mine safety, construction, safety performance, safety production, coal mine, intrinsic safety, and AHP fuzzy comprehensive evaluation.

In contrast to domestic research, the prominence of keywords on the international timeline starts later and changes more gradually, undergoing three stages of slow development, rapid growth, and decline. In the graph, nodes related to safety performance, safety management, and safety behavior are more prominent, indicating that these keywords are research hotspots in the field of international enterprise safety culture. Analyzing each stage, during the slow development stage, scholars mainly explored the construction of safety culture within enterprises and the impact of the formation of a safety atmosphere on employee behavior (14, 15). In the rapid growth stage, scholars further expanded the influence of leaders and senior management on enterprise safety culture and safety performance based on previous research (16, 17). Finally, during the stage of declining growth, the main research focus is on the construction of safety culture and the related research on safety management systems in different countries, industries, and enterprises (18). On the right side of the graph are keyword cluster labels for foreign enterprise safety culture research, evolving into seven clusters: prospective safety performance evaluation, steel industry, applied research, production work, senior management, safety management system, and commercial transportation.

### 3.4 Burst analysis of keywords

Burst analysis is one of the important tools in current literature content mining, reflecting active or frontier research nodes. Research frontier trends are understood by detecting the burst rate of relevant literature in a certain period and tracking the future direction of research based on the changing trend of literature. Keeping other parameters unchanged, setting the minimum duration for bursting (Minimum Duration) to 1 year, and running the “Citation/Frequency Burst Histor” function, lists of bursting keywords in the fields of domestic and international enterprise safety culture were generated, obtaining 20 and 10 burst keywords, respectively. Burst words in CiteSpace refer to keywords that suddenly appear in a research field, representing the forefront of research in that field. By using the burst analysis function in CiteSpace software, this study obtained burst keywords at different stages of the literature collection.

**Figure 5:**
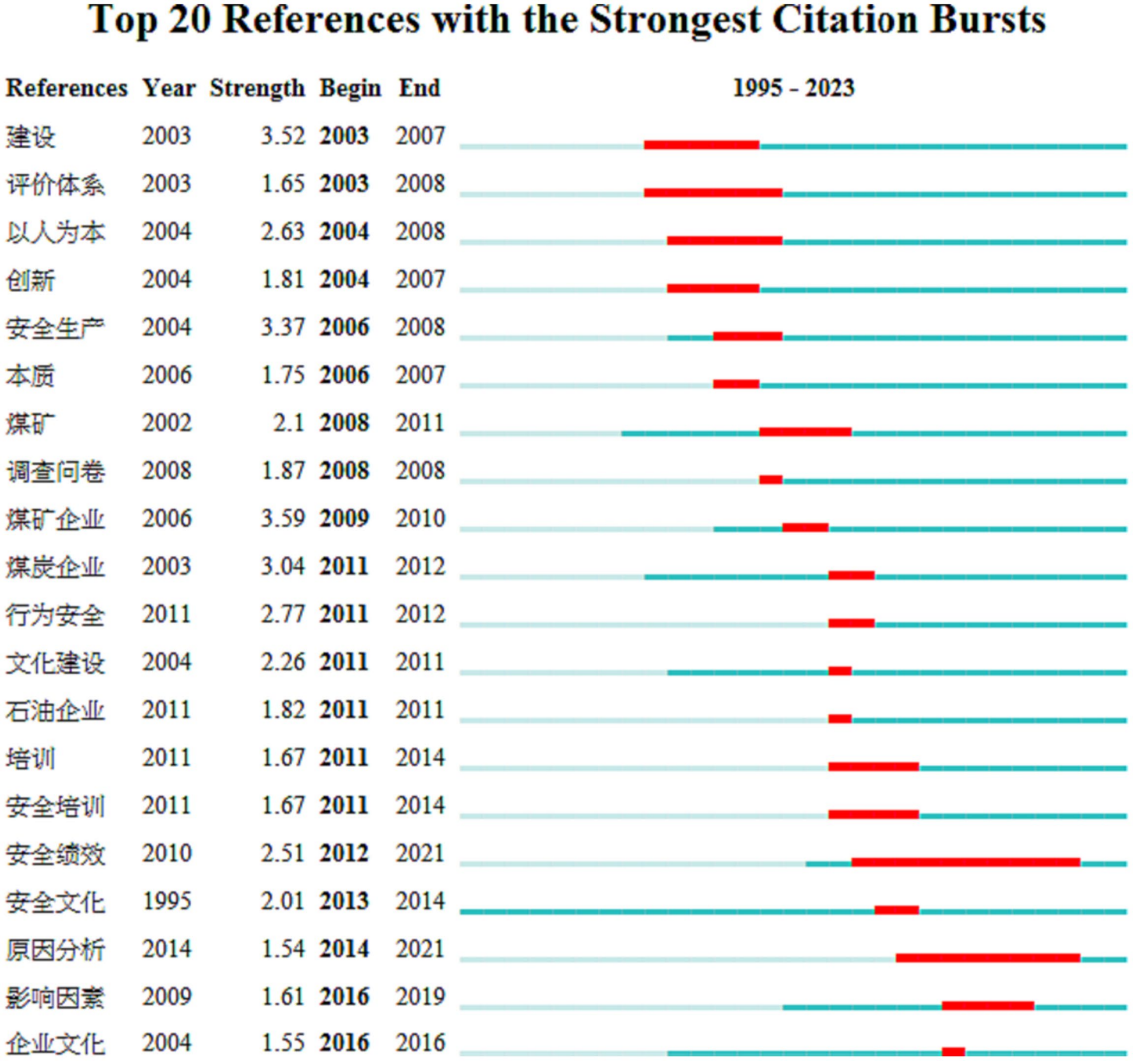
Top 20 references with the strongest citation bursts on domestic enterprise safety culture research from 1995 to 2023. They are “construction, evaluation system, people-oriented, innovation, safety production, intrinsic, coal mine, questionnaire, coal mine enterprise, behavior safety, culture construction, oil enterprise, training, safety training, safety performance, safety culture, cause analysis, influencing factors, enterprise culture”.

Utilizing CiteSpace V’s functionalities and analysis, where deeper colors indicate that researchers in the field have conducted relevant studies in that year and lighter colors indicate that almost no scholars have been involved in the field during that year. The first year of deep color appearance indicates the time when the keyword was first mentioned by scholars in the related field. In terms of domestic research results, the field of enterprise safety culture research has undergone nearly two decades of development and evolution. The results of the 20 strongest bursting keywords are shown in Figure 5. In 2006, there was a research boom in the keyword “safety production,” focusing on safety production and construction areas, as well as research hotspots related to coal mines and coal mine enterprises. In 2011, on the basis of previous research, there was a burst of research hotspots on “safety behavior,” “cultural construction,” and “safety training.” In 2016, influencing factors and enterprise culture became research hotspots. In terms of burst intensity, safety culture began to appear in 2013, while enterprise culture began to burst in 2016. Although the attention of scholars to the field of enterprise safety culture has decreased in recent years, it indicates that safety culture and enterprise culture may still be key directions and trends for future research.

**Figure 6:**
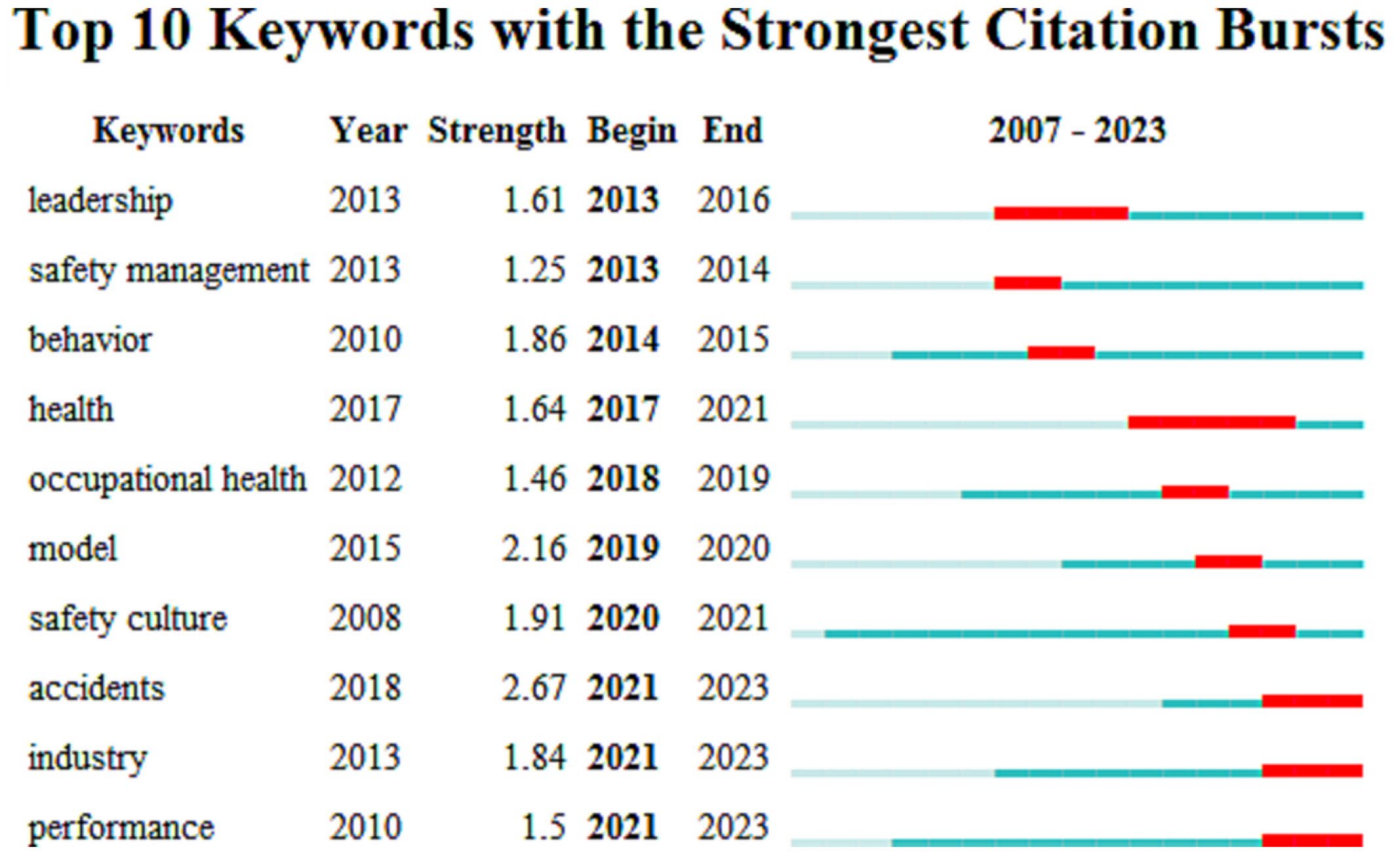
Top 10 keywords with the strongest citation bursts on international enterprise safety culture research from 2007 to 2023.

Examining the results of international research on enterprise safety culture, the field has undergone only over a decade of development and evolution. The outcomes of the ten strongest bursting keywords are presented in Figure 6. In 2013, there was a research boom regarding leadership and safety management. In 2020, there was a burst of research hotspots on safety culture based on previous research. In 2021, industrial production safety, accident prevention and handling, as well as safety performance became research hotspots. From the perspective of burst intensity, safety culture emerged after 2020, and the burst intensity of analyzing individual and organizational unsafe behaviors has been very high to the present, indicating that safety culture and accident prevention are upcoming hotspots and trends.

## 4 Discussion

This study, grounded in a bibliometric analysis of the current state of domestic and international enterprise safety culture research, alongside advancements in related research and practice, posits several areas for future research from theoretical and practical perspective.

### 4.1 Definition and system model of safety culture

Currently, a lack of a unified definition of safety culture and theory, both domestically and internationally, has led to confusion in management practices. Thus, it is imperative to precisely define enterprise safety culture and construct a unified, scientific, and systematic model. Simultaneously, a thorough analysis of various factors influencing the formation of enterprise safety culture is necessary. With the rapid development of safety accidents and accident prevention technology in recent years, there is a deeper understanding of the essence of enterprise safety culture. It is gradually recognized as a cultural atmosphere formed through long-term practical activities within a certain area. Future research can clarify the concept of enterprise safety culture by reviewing relevant theories and research findings. For example, by analyzing various types of enterprises in China to define the characteristics of their safety culture and delineate its connotations and extensions. Furthermore, future research can establish an evaluation indicator system for enterprise safety culture from the dimensions of personnel, materials, and the environment, determine the weights of various indicators using the Analytic Hierarchy Process, evaluate enterprise safety culture using fuzzy comprehensive evaluation, and propose an enterprise safety culture system model based on the dimensions of personnel and the environment.

### 4.2 Construction of enterprise safety culture system

Firstly, future research necessitates an in-depth exploration of the connotation and dimensions of enterprise safety culture. Based on this understanding, personalized enterprise safety culture systems should be crafted, accounting for the unique circumstances and industry characteristics of each enterprise. Secondly, leveraging emerging technologies, information means, intelligent devices, and systems is crucial for developing a safety culture system aligned with the specific needs of an enterprise. Different production processes, such as mechanical manufacturing or coal mining, have distinct functional requirements for the human-machine-environment system, directly influencing the role it plays during production. Therefore, constructing the enterprise safety culture system requires an analysis and study of the human-machine-environment system, ensuring it serves a more reasonable purpose. Simultaneously, research should delve into the mechanisms of the role of safety culture in various production processes and its influencing factors. Finally, future research can explore the construction of an enterprise safety culture system with Chinese characteristics. On one hand, by learning from the successful experiences and theoretical achievements of developed Western countries and combining them with China’s national conditions and enterprise realities, it’s possible to explore safety culture systems suited to China’s unique circumstances. On the other hand, by drawing on the successful experiences and theoretical achievements of advanced enterprises and combining them with the current situation of Chinese enterprises, constructing an enterprise safety culture system that aligns with China’s national conditions is essential.

### 4.3 Practical application of enterprise safety culture

Enterprise safety culture is the soul and core of safety management, constituting an essential component of safety production management. This study conducted a comprehensive literature analysis on the field of safety culture through diversified, time-sharing, and dynamic information visualization technologies. Through analysis, the distribution of research hotspots, trends, and research forces in the field of safety culture can be clearly presented. The results show that despite extensive safety culture-related research, several issues persist, including a lack of unified understanding of safety culture definitions, essence, and developmental paths, limited research methods, and a prevalence of qualitative research over quantitative research. Therefore, practitioners should aim for a comprehensive understanding of safety culture and guide theoretical development through practical applications. In addition, this study also reveals key research issues and gaps in the field of safety culture, which may become issues that practitioners need to pay attention to and solve in practical work, thereby promoting continuous improvement and refinement of safety culture practices. The research results of this article indicate that practitioners in enterprises should systematically and deeply study the main factors and related mechanisms that affect safety culture. They should pay attention to the development of evaluation and evaluation mechanisms, innovation and promotion models for enterprise safety culture. At the same time, it is crucial to establish a standardized evaluation index system to adapt the construction of enterprise safety culture to the characteristics of the enterprise and the actual production process. Furthermore, effective models for the practical application of safety culture should be explored based on the actual situation of different industries, enterprises, and regions.

## 5 Conclusion

Building upon a systematic examination of domestic and international literature on enterprise safety culture, this research, through a comprehensive literature analysis, precisely delineated the concept and theoretical underpinnings of enterprise safety culture. Conducting an in-depth content analysis of 635 domestic and international documents, this study discerned the distribution of research themes pertaining to enterprise safety culture. It was observed that domestic research predominantly centers on the practical perspective of mitigating accidents through the establishment of enterprise safety culture, while international research places greater emphasis on theoretical considerations, specifically focusing on the impact of safety culture atmospheres within enterprises on employees. These findings offer valuable insights for the trajectory of domestic enterprise safety culture research.

Subsequently, employing literature visualization analysis, this study outlined the domestic and international research hotspots and trends in enterprise safety culture. It surfaced that, in contrast to international scholars engaging in theoretical discussions, domestic research predominantly concentrates on defining the concept of enterprise safety culture, categorizing dimensions, scrutinizing influencing factors, and appraising effects. Given the escalating severity of safety production situations, there is a growing urgency for enterprise safety culture research in China. However, there exists a relative scarcity of research on the factors influencing enterprise safety culture and its effectiveness, necessitating further empirical analysis.

In conclusion, recognizing the limitations in existing domestic and international research, this study suggests further avenues for the advancement of enterprise safety culture research. These include refining the definition of the safety culture concept and system model, developing the enterprise safety culture system, exploring the practical application of enterprise safety culture, and examining the interplay between enterprise safety culture and pertinent national laws, regulations, and policies.

Compared with existing literature, this study uses CiteSpace to conduct a systematic analysis of safety culture related research. The uniqueness of this article lies in the following three aspects: firstly, it provides a comprehensive research perspective. By integrating and analyzing a large number of literature, this article provides researchers with a comprehensive perspective on enterprise safety culture, which helps them to have a deeper understanding of the complexity and diversity of this field; Secondly, revealing research gaps and trends, this article reveals the research gaps and trends in the field of corporate safety culture through visual knowledge graphs, providing valuable references for future research; Thirdly, promoting interdisciplinary cooperation. The analysis results of this article can help researchers from different disciplines find common research interests, thereby promoting interdisciplinary cooperation and communication. In summary, this article not only provides researchers with deeper insights, but also provides more specific guidance and suggestions for practitioners.

In the future research on safety culture, on the one hand, it is possible to deeply analyze the application of artificial intelligence and machine learning in safety culture. With the rapid development of artificial intelligence and machine learning, these technologies are becoming increasingly widely used in the field of safety. For example, these technologies can be used to predict and prevent accidents, improve safety performance, optimize safety processes, etc. Therefore, further research is needed on how to better integrate these technologies into safety culture to improve safety levels and efficiency. On the other hand, research on cross-cultural safety culture can be widely carried out. In the context of globalization, communication and interaction between different cultures are becoming increasingly frequent. Therefore, further research is needed on the differences and similarities of safety culture in different cultural backgrounds in order to better understand and respond to cross-cultural safety issues.

## Author contributions

MW: Formal analysis, Investigation, Methodology, Resources, Visualization, Writing – original draft, Writing – review & editing. ZG: Formal analysis, Investigation, Resources, Writing – original draft, Writing – review & editing. CX: Formal analysis, Investigation, Methodology, Resources, Writing – original draft, Writing – review & editing. YW: Formal analysis, Investigation, Methodology, Resources, Writing – original draft, Writing – review & editing. DC: Formal analysis, Investigation, Resources, Writing – original draft, Writing – review & editing.
